# Alterations in Rumen Bacterial Community and Metabolome Characteristics of Cashmere Goats in Response to Dietary Nutrient Density

**DOI:** 10.3390/ani10071193

**Published:** 2020-07-14

**Authors:** Yaoyue Wang, Peng Tang, Yafei Xiao, Jianming Liu, Yulin Chen, Yuxin Yang

**Affiliations:** College of Animal Science and Technology, Northwest A&F University, Yangling 712100, China; yaoyue@nwafu.edu.cn (Y.W.); tangpeng2020@nwafu.edu.cn (P.T.); pointyafei@nwafu.edu.cn (Y.X.); liujianming321@nwafu.edu.cn (J.L.)

**Keywords:** dietary energy and protein, metabolomics, rumen bacterial composition, Shaanbei white cashmere goats, 16S rRNA gene sequencing

## Abstract

**Simple Summary:**

Dietary energy and protein play important roles in rumen fermentation. However, the impacts of dietary energy and protein on the relationships between rumen bacterial composition and ruminal metabolites were not extensively studied. In this study, we investigated the rumen fermentation status in response to different energy and protein levels, which was vital for the rumen health and resulted in the potential positive effects on the host health and production. Thereby, quantitative polymerase chain reaction analysis and 16S rRNA gene sequencing showed that the bacterial richness was significantly reduced, and the rumen bacterial composition was significantly altered with the increasing levels of dietary energy and protein. Metabolomics analysis revealed that the dominant differential metabolites were amino acids, peptides, and analogs. Moreover, high-energy and high-protein diets could enhance the ruminal antioxidative capacity by increasing the concentration of some metabolites. Correlation analysis implied that high energy and protein levels could enhance the catechol concentration by *Prevotella_*1 and *Ruminococcus*_2. This study can help to improve the dietary energy and protein use efficiency in goats.

**Abstract:**

This study was conducted to investigate the impacts of dietary energy and protein on rumen bacterial composition and ruminal metabolites. A total of 12 ruminal samples were collected from Shaanbei white cashmere goats which were divided into two groups, including high-energy and high-protein (Group H; crude protein, CP: 9.37% in dry matter; metabolic energy, ME: 9.24 MJ/kg) and control (Group C; CP: 8.73%; ME: 8.60 MJ/kg) groups. Thereby, 16S rRNA gene sequencing and a quantitative polymerase chain reaction were performed to identify the rumen bacterial community. Metabolomics analysis was done to investigate the rumen metabolites and the related metabolic pathways in Groups C and H. The high-energy and high-protein diets increased the relative abundance of phylum Bacteroidetes and genera *Prevotella*_1 and *Succiniclasticum*, while decreasing the number of Proteobacteria (*p* < 0.05). The dominant differential metabolites were amino acids, peptides, and analogs. Tyrosine metabolism played an important role among the nine main metabolic pathways. Correlation analysis revealed that both *Prevotella*_1 (*r* = 0.608, *p* < 0.05) and *Ruminococcus*_2 (*r* = 0.613, *p* < 0.05) showed a positive correlation with catechol. Our findings revealed that the diets with high energy and protein levels in Group H significantly altered the composition of ruminal bacteria and metabolites, which can help to improve the dietary energy and protein use efficiency in goats.

## 1. Introduction

Shaanbei white cashmere goat (SWCG) is a local breed in the northern Shaanxi province of China and the total population of SWCG exceeds 10 million. SWCG is well-known for cashmere wool and meat, which are the most important economic sources of the local farmers [1,2]. Traditional grazing management is mainly dependent on natural pastures, which are limited in the extremely harsh winter. Hence, nutritional management, especially the choice of dietary nutrient density, is important to promote the growth of goats [3].

The rumen is a complex microbial ecosystem in ruminants. It can ferment feedstuffs to volatile fatty acids, microbial proteins, and vitamins, which play important roles in animal health and production [4,5]. Sometimes, it has to deal with lower quality fodder or silage [6]. Among the microbiota, bacteria are the most abundant, diverse, and metabolically active species in the rumen [7,8]. The bacterial community in the rumen are linked to various factors, such as animal diet, breed, age health and geographic region [9,10]. Diet is the major determinant of the microbial composition in the rumen [11]. For example, fibers could be degraded into glucose and xylose by *Ruminococcus* [12].

The functions of the rumen microbiota make ruminants highly adaptable to various diets [13]. The energy and protein in the diets are the most restrictive factors for ruminal microbial growth [14,15]. Dietary protein is utilized to synthesize microbial crude protein (MCP) for host utilization by Bacteroidetes and *Prevotella* [16,17]. However, protein overfeeding increases the excreted nitrogen from urine and feces, which causes environmental pollution [18] and economic losses [19]. Previous studies have reported that dietary energy can promote protein to synthesize MCP [20,21]. The effective way to improve production performance in cattle is increasing dietary energy levels under the same concentration of forage ratio [22]. 

Previous studies showed that the phenotypic traits of ruminants were affected by rumen microbiota, whose functions could be reflected by the ruminal metabolites [23]. However, most of the studies have only focused on the change in dietary energy [3,20] or protein [16,18], and few reports studied the effects of dietary energy and protein on the rumen bacterial composition and rumen metabolites. 

In our previous study, we detected that high levels of dietary energy (metabolic energy, ME: 9.24 MJ/kg) and protein (crude protein, CP: 9.37% in dry matter (DM)) could significantly enhance the average daily weight gain (ADG), dressing percentage, and eye muscle area of SWCG [24]. The primary objective of this study was to investigate the changes in the rumen bacterial diversity by 16S rRNA gene sequencing and quantitative polymerase chain reaction (qPCR), and the metabolites and key metabolic pathways by gas chromatography tandem time-of-flight mass spectrometry (GC-TOFMS)-based metabolomics by increasing dietary energy and protein. Furthermore, we examined the relationships between ruminal bacterial communities and rumen metabolites to improve dietary energy and protein use efficiency in SWCG.

## 2. Materials and Methods

### 2.1. Ethics Statement

The use of animals and all experimental protocols (protocol number: 100403) were authorized by the Institutional Animal Care and Use Committee of Northwest A&F University (Yangling, Shaanxi, China).

### 2.2. Animals, Diets and Sampling

A total of 12 SWCG (age of 8 months, an average initial body weight of 24.5 ± 1.87 kg, six males and six females) were selected and fed at Diqingyuan farm (37.6° N, 108.79° E), Yulin, Shannxi Province, China. All goats were randomly allocated into two treatments based on their body weights (3 males and 3 females in each group). Each goat was housed in an individual pen, and the goats were fed individually. The diets were formulated based on the Feeding Standard of Meat-Producing Sheep and Goats (NY/T816-2004, China). Group H included high energy (metabolic energy, ME: 9.24 MJ/kg) and high protein (crude protein, CP: 9.37% in dry matter) diet. Group C included the basal diet (CP: 8.73%; ME: 8.60 MJ/kg). Rumen degradable protein (RDP) in Groups C and H was 3.62% and 4.04%, respectively. Rumen degradable starch (RDS) in Groups C and H was 11.91% and 12.54%, respectively. Also, the ratio of dietary energy to protein and the ratio of dietary forage to concentrate in the two groups were not changed (Table 1). Corn stalk and alfalfa were used to adjust the concentrate: forage ratio and dietary energy: protein ratio.

All animals were fed at 9:00 am and 4:00 pm daily. The experiment lasted for 65 days, including 10 days for acclimation period. Average daily feed intakes (ADFI) of per goat in Groups C and H was 1.13 kg/d and 1.14 kg/d, respectively. Hence, the ADFI of CP for each sheep in Groups C and H was 98.6 g and 106 g, respectively. After slaughtering the goats, the rumen was opened from the ventral sac. The rumen content of each goat was first homogenized by hand using sterile gloves, and then approximately 50 mL contents were collected. Rumen sample of each goat was individually filtered through four layers of sterile cheesecloth to separate rumen fluid from rumen solid phase. Then, the liquid samples were immediately frozen in liquid nitrogen and stored at −80 °C for further studies.

### 2.3. DNA Extraction, 16S rRNA Gene Amplicon and Sequencing

Bacterial genomic DNA was extracted from rumen fluid samples using a stool DNA kit (OMEGA Bio-Tek, Norcross, GA, USA). The extracted DNA was checked on 1% agarose gel, and DNA concentration and purity were determined with NanoDrop 2000 UV-vis spectrophotometer (Thermo Scientific, Wilmington, DE, USA). Total DNA from rumen content samples was used to construct 16S rRNA libraries by the NEXTflex Rapid DNA-Seq Kit (Bioo Scientific Cor. Austin, TX, USA) [25]. The universal primers [V338F (5′-ACTCCTACGGGAGGCAGCAG-3′) and V806R (5′-GGACTACHVGGGTWTCTAAT-3′)] targeting the V3-V4 region of bacteria were used. PCR products were mixed with equimolar ratios and purified by Qiagen Gel Extraction Kit (Qiagen, Dusseldorf, Germany). Finally, Illumina MiSeq platform was used for Paired-End sequencing at Majorbio Bio-Pharm Technology Co. Ltd. (Shanghai, China). 

### 2.4. Sequence Processing

The raw data were merged by FLASH (version 1.2.11) [26] and filtered to obtain the clean data by the QIIME 1.9.1 [27]. Briefly, (i) 300 bp reads were truncated at any site with an average quality sore <20 over a 50 bp sliding window; (ii) both truncated reads shorter than 50 bp or containing ambiguous characters were discarded; (iii) overlapping sequences with at least a 10 bp overlap were assembled and the maximum mismatch ratio of overlap region was 0.2; (iv) samples were distinguished by the barcodes and primers. Barcode allowed mismatch was 0 and the maximum nucleotide mismatch in primer was 2. Operational taxonomic units (OTUs) were clustered with 97% similarity cut-off using UCLUST [28]. The indices of Alpha diversity were analyzed by MOTHUR (version v.1.30.2) [29]. Beta diversity was calculated by weighted UniFrac distance. Rarefaction curves, Venn diagram, bar and heatmap graphs were visualized by R software (version 3.1.2). The statistical significance of grouping was assessed by the analysis of similarity (ANOSIM) in QIIME 1.9.1 and it was determined by calculating *p*-value with 999 permutations [29,30]. Significant interactions between rumen bacteria were shown using Networkx. The raw reads have been deposited in the National Center for Biotechnology Information (NCBI) Sequence Read Archive (SRA) (Accession number: SRP258202).

### 2.5. Prediction of Rumen Bacterial Function

The function of detected rumen bacteria was predicted via the phylogenetic analysis of communities by reconstruction of unobserved states (PICRUSt2) [31]. The predicted sequences were aligned to the Kyoto Encyclopedia of Genes and Genomes (KEGG) database [32].

### 2.6. qPCR Analysis between Different Groups

Changes in phylum Bacteroidetes and genus *Prevotella* between different treatments were verified by absolute qPCR using iCycle thermos cycler (Bio-Read-CFX, CA, USA). The primers and annealing temperature were shown in Table S1. The external standard curves were constructed by 10-fold serial dilution of plasmid DNA containing the cloned marker loci. All standard curves met the required efficient standards (R^2^ > 0.99, 90% < E < 120%). The reaction mixture and conditions were according to our previous study [33].

### 2.7. Metabolomics Analysis by GC-TOFMS

The ruminal fluid was used for metabolomics analysis. For each sample, 200 μL rumen fluid sample, 200 μL liquid methanol and 20 μL L-2-Chlorophenylalanine (CAS#: 103616-89-3, ≥98%) (1 mg/mL in H_2_O) as an internal standard were sequentially added to the 1.5 mL Eppendorf (EP) tubes. The mixture was vortexed for 10 s and then centrifuged at 13,000 rpm for 15 min at 4 °C. After centrifugation, 370 μL of the supernatant was transferred to a 2 mL GC/MS glass vial and dried in a vacuum concentrator without heating. After evaporation, 80 μL of methoxy amination hydrochloride (20 mg/mL in pyridine) was added to the sample and incubated for 30 min at 80 °C. Meanwhile, 100 μL of the N,O-Bis (trimethylsilyl) trifluoroacetamide (BSTFA) with 1% trimethylchlorosilane (TMCS) was added to the sample aliquots. The mixture was incubated at 70 °C for 1.5 h. 

Derivatized samples were analyzed using the Agilent 7890B gas chromatograph system (Agilent, Santa Clara, CA, USA) coupled with the LECO Chroma TOF PEGASUS HT (LECO, St. Joseph County, MI, USA) [34]. Injecting 1 μL aliquot of the analyte into splitless mode, helium was used as the carrier gas. The injection, transfer line and ion source temperatures were 280, 270, and 220 °C, respectively. The mass spectrometry data were performed in full-scan mode with 50–500 m/z at 20 scans/s after 6.1 min of solvent delay. 

The extraction of raw peaks, filtering and calibration of the baseline data, peak alignment, deconvolution analysis, peak identification, and peak area integration were performed by Chroma TOF 4.3X software [35]. The content of each component was calculated by the peak area normalization method. Principal component analysis (PCA) and orthogonal correction partial least squares discriminant analysis (OPLS-DA) were conducted using SIMCA software (V14.1). 

### 2.8. Statistical Analysis

Statistical analysis was performed by Statistical Package for the Social Sciences (SPSS) version 20.0 (SPSS Inc., Chicago, IL, USA). Significant differences between Groups C and H were calculated using the Student’s *t*-test (for normally distributed data) and the Mann–Whitney *U* test (for non-normally distributed data). For the GC-TOFMS data, differential metabolites between two groups were identified combining variable importance in the projection (VIP) >1 and *p* < 0.05. Significant correlations between rumen bacteria and metabolite variables were assessed by Spearman correlation analysis, if the correlation coefficients (r, in absolute values) were above 0.55 [36,37]. The statistical significance was set at *p* < 0.05. 

## 3. Results

### 3.1. Diversity, Richness and Similarity of the Ruminal Bacterial Communities

**Figure 1:**
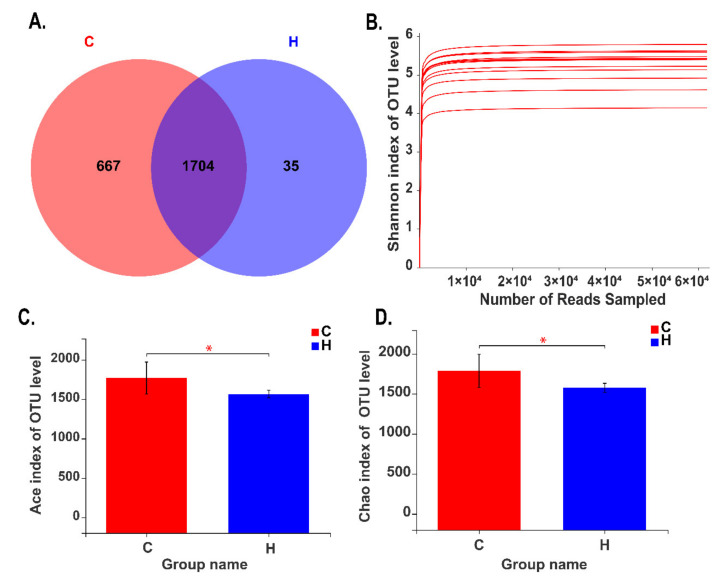
16S rRNA gene sequences in different dietary groups. A Venn diagram illustrating the overlap of bacterial OTUs at a 3% dissimilarity level for Groups C and H. (**A**). The samples of Group C included the goats fed with a typical total mixed ration (TMR), and the samples of Group H included the goats fed with the high energy and high protein diets. Rarefaction analysis of different samples (**B**). Differences in ACE indices (**C**) and Chao indices (**D**) between Groups C and H.

A total of 826,727 16S rRNA gene sequences were obtained from 12 different samples with 61,658 rarefied sequencing reads per sample after quality control and chimaera removal. In this study, the average sequences were 68,110 ± 4362 (minimum: 61,658; maximum: 71,473) in Group C and 69,677 ± 3532 (minimum: 67,740; maximum: 72,873) in Group H. Additionally, there was no significant difference in sequencing reads between Groups C and H. Group C exhibited the highest number of unique sequences (667 OTUs), followed by Group H (35 OTUs). Approximately 71% of the total OTUs (1704 OTUs) were shared by two groups (Figure 1A). The rarefaction curves (Figure 1B) reached the saturation plateau and the indices of Good’s coverage were above 0.99 (Supplementary Table S2), indicating that the sequencing depth was reasonable. ACE (Figure 1C) and Chao (Figure 1D) indices were significantly decreased when the goats were fed with high energy and protein diets in Group H (*p* < 0.05), while Shannon and Simpson indices had no significant effects (*p* > 0.05) (Supplementary Table S2).

ANOSIM (*p* < 0.05) was performed to indicate the statistical difference between the treatments. Table 2 showed that the rumen bacterial community structures at phylum (*p* = 0.037), genus (*p* = 0.009), and OTU levels (*p* = 0.014) were significantly different between Groups C and H. 

### 3.2. Composition and Differences of Ruminal Bacterial Communities

**Figure 2:**
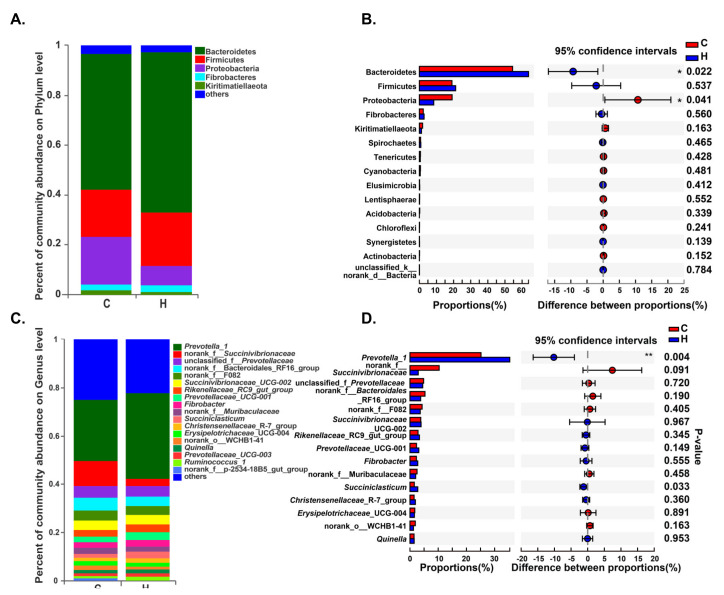
Distribution of bacteria in different groups. The color-coded bar plots represent the average distribution of bacterial phyla (**A**) and genera (**C**), respectively. Only the dominant bacteria (with a relative abundance ≥1%) among rumen bacteria are shown. Extended error bar plots illustrate the mean proportions and differences in the phyla (**B**) or genera (**D**) in rumen samples. * indicates *p* < 0.05.

A total of 30 phyla were detected by taxonomic analysis. The top five prominent phyla in Groups H and C were Bacteroidetes (abundances of 63.76% and 54.46%, respectively), Firmicutes (21.20% and 19.04%), Proteobacteria (8.40% and 19.13%), Fibrobacteres (2.76% and 2.29%), and Kiritimatiellaeota (1.24% and 1.90%), which are accounted for more than 96% (Figure 2A, Supplementary Table S3). With the increase of energy and protein levels in diets, the abundance of Bacteroidetes increased significantly (*p* < 0.05), while the abundance of Proteobacteria significantly decreased (*p* < 0.05) (Figure 2B, Supplementary Table S3). 

When sequences were analyzed at a lower taxonomical level, more detailed information about rumen bacteria was found. A total of 539 bacterial genera were detected. Within Group C, the most abundant sequences were those related to *Prevotella*_1 (abundance of 25.17%), norank_f__*Succinivibrionaceae* (10.35%), norank_f__*Bacteroidales_*RF16_group (5.33%), unclassified_f__*Prevotellaceae* (4.85%), norank_f__*F082* (4.31%) and *Succinivibrionaceae_*UCG-002 (3.84%). Within Group H, the dominant taxa were associated with *Prevotella*_1 (35.36%), unclassified_f__*Prevotellaceae* (4.53%) *Succinivibrionaceae_*UCG-002 (3.94%), norank_f__*Bacteroidales_*RF16_group (3.79%), norank_f__*F082* (3.64%), and *Rikenellaceae*_RC9_gut_group (3.36%) (Figure 2C, Supplementary Table S4). In addition, the relative abundances of genera *Prevotella*_1 and *Succiniclasticum* were significantly increased when energy and protein levels in diets were increased (*p* < 0.5) (Figure 2D, Supplementary Table S4).

### 3.3. qPCR Analysis

According to 16S rRNA gene sequencing data, the differences in the number of Bacteroidetes (phylum level) and *Prevotella* (genus level) between Groups C and H were further verified by absolute qPCR. As shown in Table 3, the number of *Prevotella* and Bacteroidetes in the rumen of Group H was significantly increased (*p* < 0.05) compared with Group C. 

### 3.4. Functional Predictions of Rumen Bacteria

**Figure 3:**
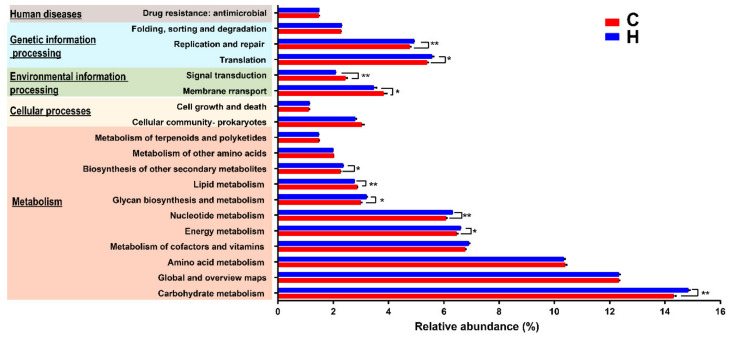
Differences in bacterial metabolism function at KEGG level 2 between Groups C and H using PICRUSt2.

The potential functions of the bacterial community in the rumen of SWCG were predicted by the PICRUSt2 based on 16S rRNA gene sequencing data. At KEGG level 1, metabolism-related pathways had the highest abundance (>50%). Compared with Group C, the rumen bacteria of Group H was predicted to have significantly higher capability of influencing genetic information processing and lower capability of influencing environmental information processing and human disease (*p* < 0.05) (Supplementary Table S5). At KEGG level 2, the highest relative abundance was carbohydrate metabolism. In addition, the abundances of genes belonged to carbohydrate metabolism, energy metabolism, nucleotide metabolism, glycan biosynthesis and metabolism, biosynthesis of other secondary metabolites, translation, and replication and repair were significantly higher in Group H than those in Group C. The abundances of genes involved in lipid metabolism, membrane transport, and signal transduction were significantly higher in Group C compared with Group H (Figure 3, Supplementary Table S6).

### 3.5. Metabolic Phenotype Profile of Rumen

**Figure 4:**
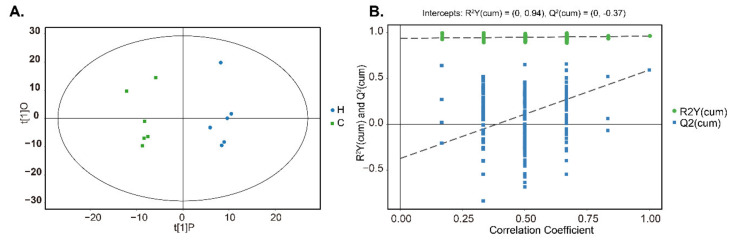
Orthogonal partial least squares discriminant analysis [(O)PLS-DA] plots of rumen metabolites between Groups C and H. Score scatter plot of OPLS-DA model for Group H versus C **(A)**. Permutation test of OPLS-DA model for Group H versus C **(B)**.

A total of 607 valid peaks were integrated after GC-TOFMS analysis of rumen contents and the chromatograms were shown in Supplementary Figure S1A. Among these peaks, 264 metabolites were identified according to their major chemical classes, including amino acids, peptides, and analogues, fatty acids and conjugates; dicarboxylic acids and derivatives, and phenols and derivatives (Table 4, Supplementary Table S7). A global overview of the significant differences among the metabolites was first examined by PCA (Supplementary Figure S1B). These differences were further verified by the score plots of OPLS-DA (Figure 4A). The values of R^2^Y (0.94) and Q^2^intercept (−0.37) indicated the robustness of the OPLS-DA models (Figure 4B). 

### 3.6. Differences in the Ruminal Metabolites between Groups C and H 

Twenty-four differential metabolites between Groups C and H were identified using VIP analysis and Student’s *t*-test (VIP > 1, *p* < 0.05) (Table 3). The main differences in ruminal metabolites between Groups C and H were the variation of amino acids, peptides, and analogs; pyridine; fatty acids and conjugates; lipids and lipid-like molecules; sugars; sugar acids and derivatives; amines. Among the metabolites, the levels of uracil, itaconic acid, 5-methoxyindole-3-acetic acid, methyl *trans*-cinnamate, spermidine, and catechol were higher in Group H compared with Group C. The concentrations of the remaining sixteen metabolites decreased significantly with the increase of dietary energy and protein levels. 

### 3.7. Metabolic Pathways of Differential Metabolites

**Figure 5:**
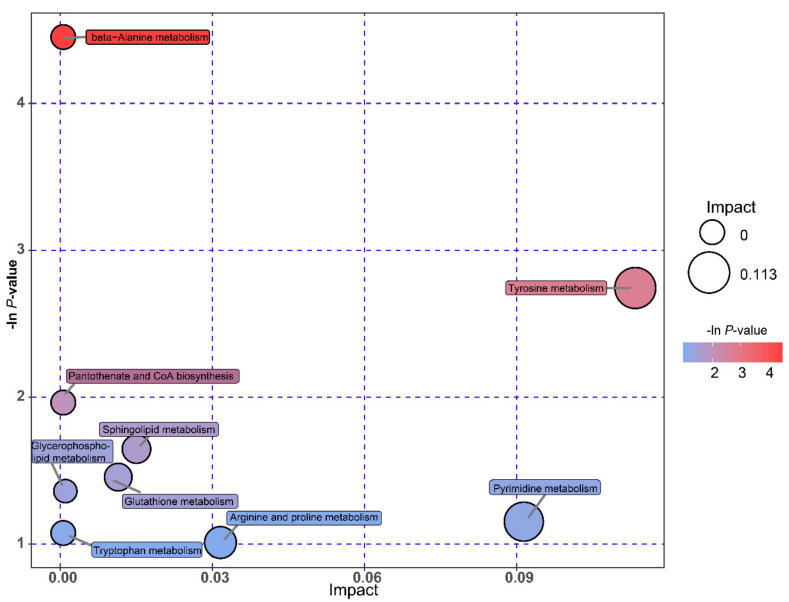
Metabolome view map of the deferential metabolites (VIP > 1, *p* < 0.05) identified in the rumen from the goats fed with the diets with different energy and protein levels. The large size indicates high pathway enrichment, and dark color indicates high pathway impact values.

Pathway analysis is visualized in Figure 5. The varied rumen microbial metabolites between Groups C and H were identified to be mainly involved in the nine main metabolic pathways, including beta-alanine metabolism; tyrosine metabolism; pantothenate and CoA biosynthesis; sphingolipid metabolism; glutathione metabolism; glycerophospholipid metabolism; pyrimidine metabolism; tryptophan metabolism; arginine and proline metabolism. These pathways are mainly involved in amino acids metabolism, lipid metabolism, and nucleotide metabolism. Additionally, among these metabolic pathways, tyrosine metabolism had the largest impact.

### 3.8. Correlation Analysis between Rumen Bacteria and Rumen Metabolites

Based on Spearman correlation analysis (|*r*| > 0.55 and *p* < 0.05), we constructed the correlation networks between the bacterial genera in Groups C and H, respectively. As shown in Figure S2A and Figure S2B, 171 and 79 edges were observed in Groups C and H, respectively, which indicated that the relationships between the bacterial genera in Group C were more complex than those in Group H. The comprehensive relationships between ruminal bacterial genera were observed in this study (Table S8). Of these, *Prevotella*_1 was positively correlated with *Succiniclasticum* (*r* = 0.580, *p* < 0.05) and *Ruminococcus*_2 (*r* = 0.651, *p* < 0.05). *Selenomonas*_1 was positively correlated with *Prevotellaceae*_UCG-004 (*r* = 0.78, *p* < 0.01)

**Figure 6:**
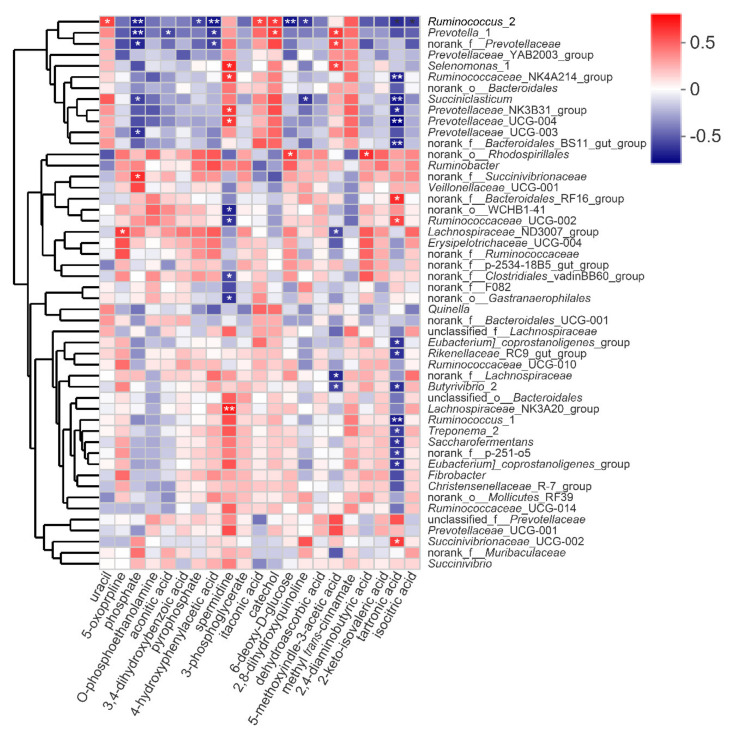
Correlation between bacterial genera and deferential metabolites affected by diets with different energy and protein levels. Correlations are indicated by red and blue colors; 1 indicates a perfectly positive correlation (dark red) and close to −1 indicates a perfectly negative correlation (dark blue). Weak correlation is indicated by white colors. * and ** indicate *p* < 0.05 and *p ≤* 0.01, respectively.

We determined the relationships between the differential metabolites and the top 50 bacterial communities at the genus level (Figure 6 and Supplementary Table S9). *Prevotella_*1 was positively correlated with 5-methoxyindole-3-acetic acid (*r* = 0.601, *p* < 0.05) and catechol (*r* = 0.608, *p* < 0.05), but negatively correlated with aconitic acid (*r* = −0.594, *p* < 0.05), 4-hydroxyphenylacetic acid (*r* = −0.643, *p* < 0.05) and phosphate (*r* = −0.720, *p* < 0.01). *Succiniclasticum* was negatively correlated with phosphate (r = −0.62, *p* < 0.05) and 2,8-dihydroxyquinoline (*r* = −0.65, *p* < 0.05). *Ruminococcus*_2 was positively correlated with uracil (*r* = 0.578, *p* < 0.05), catechol (*r* = 0.613, *p* < 0.05) and itaconic acid (*r* = 0.578, *p* < 0.05), while negatively correlated with 4-hydroxyphenylacetic acid (*r* = −0.75, *p* < 0.01). In addition, 5-oxoproline had high positive correlation with *Lachnospiraceae*_ND3007_group (*r* = 0.608, *p* < 0.05). Also, spermidine was positively correlated with *Selenomonas*_1 (*r* = 0.678, *p* < 0.05), *Ruminococcaceae*_NK4A214_group (*r* = 0.629, *p* < 0.05), *Lachnospiraceae*_NK3A20_group (*r* = 0.722, *p* < 0.01), *Prevotellaceae_*UCG−004 (r = 0.615, *p* < 0.05) and *Prevotellaceae_*NK3B31_group (*r* = 0.615, *p* < 0.05), while was negatively correlated with norank_*Gastranaerophilales*(r = −0.657, *p* < 0.05), norank_*Clostridiales*_vadinBB60_group (*r* = −0.601, *p* < 0.05), norank_WCHB1-41 (*r* = −0.706, *p* < 0.05) and *Ruminococcaceae*_UCG−002 (*r* = −0.650, *p* < 0.05). Both *Butyrivibrio*_2 and norank_*Lachnospiraceae* were negatively correlated with 5-methoxyindole-3-acetic acid (*r* = −0.606, *p* < 0.05; *r* = −0.685, *p* < 0.05), respectively.

## 4. Discussion

### 4.1. Comparison of the Composition and Differences of Ruminal Bacterial Communities

Diets with high energy and protein levels in Group H effectively promoted the growth performance and carcass characteristics of goats [24]. Meanwhile, the digestion and absorption of these diets were closely related to the rumen bacteria [5]. A previous study reported that the changes in the ruminal microbiota could promote the ADG of goats [22]. Therefore, we determined the differences of rumen bacterial communities of SWCG with simultaneous changes in dietary energy and protein levels in this study. 

In this study, 16S rRNA gene sequencing was used to assess the rumen bacterial community in SWCG. Liu et al. [38] and Tapio et al. [39] reported that the richness of the bacterial community was influenced by diets. Our results also revealed low bacterial richness (ACE and Chao indices) with the increasing levels of dietary energy and protein, while no significant changes were detected in the bacterial diversity (Shannon and Simpson indices). In line with the previous studies [40,41], this study revealed that Bacteroidetes, Firmicutes, and Proteobacteria were the most dominant phyla in the two groups. These bacterial phyla were the core microbiota in the rumen and their structural compositions were unchanged regardless of feeding different types of diets [42]. Among the thirty phyla, the abundance of Bacteroidetes increased significantly in Group H, which might be related to the protein degradation function of this phylum [43] and the high level of RDP in Group H. 

The effects of dietary nutrient density on the bacterial population at the genus level were also detected in this study. Similar to the results of decreased bacterial richness in Group H, the high energy and protein levels of diets reduced the complexity of rumen bacterial interactions. Among the genera, *Prevotella*_1, belonging to the Bacteroidetes, was the most abundant bacteria in both the rumens of goats fed with different diets, which was consistent with the previous reports [3,22]. In addition, the increased population of *Prevotella*_1 in Group H, might be associated with the starch and protein degrading function of this genus [3,17] and the higher RDS and RDP levels in Group H. This result was also in line with the study of Wang, et.al. [3] who reported that the number of *Prevotella*_1 was increased when the animals fed with the high protein diet. When ruminants were fed with the high-energy diets rich in starch, the increasing number of *Succiniclasticum* stabilized the rumen environment by degrading succinate to propionate [44], which might be the reason for the higher number of *Succiniclasticum* in Group H with higher RDS levels. Bacteroidetes and *Prevotella* were selected in this study to verify the differences between two groups by absolute qPCR and the results agreed with those by 16S rRNA gene amplicon sequencing. Furthermore, the previous studies have reported that the level of dietary protein was positively correlated with the relative abundance of *Prevotella* [45,46] and the increasing levels of protein could promote the growth of cellulolytic bacteria [18]. Hence, the cellulolytic bacteria-*Succiniclasticum* and *Ruminococcus*_2 [12] were positively associated with *Prevotella*_1 in our study. The differences in rumen bacteria in this study suggested that high energy and protein levels in the diets might increase the number of protein-degrading bacteria.

### 4.2. Functional Prediction of the Ruminal Bacteria in SWCG

Whether the changes in the bacterial community structures would lead to functional differences were detected by PICRUST2. Consistent with Miao et al. [47] and He et al. [48], we found that the abundances of metabolism were the highest in the rumen at KEGG level 1. The different abundances of environmental information processing and genetic information processing between two treatments suggested that varying levels of energy and protein in the diets could influence the biochemical processes of ruminal bacteria in goats at molecular and cellular levels [49]. Liu et al. [50] also reported that the KEGG pathways involved in carbohydrate metabolism were highly enriched in the microbiota of individuals fed with high energy diets, which was consistent with our study. Furthermore, the increasing number of *Prevotella*_1 in Group H of this study and the involvement of the genus in energy metabolism, nucleic acid metabolism and glycan biosynthesis and metabolism [46] might indicate the increase of the above pathways in Group H. Although the PICRUSt2 approach was utilized to predict the rumen bacterial functions, this method did not accurately detect the related functions due to the limited number of sequencing studies in ruminants [51].

### 4.3. Comparison of the Composition and Differences of Ruminal Metabolites

Microbiota interacts with numerous physiological functions in the host through its metabolic products [52]. Thus, we used the GC-TOFMS analysis to explore the metabolic functions of ruminal microbiota. The main metabolites were amino acids, peptides, and analogues in this study, which was consistent with the previous reports [38,53]. Amino acids in the rumen are the key precursors for protein and polypeptides synthesis and are mainly obtained from the dietary proteins and microproteins [54].

According to the OPLS-DA results, a clear difference of ruminal metabolites was demonstrated between Groups C and H. These results confirmed that ruminal metabolites were closely related to the composition of diets [55]. 

Uracil concentration in the rumen was increased with the increase density of starch in diets [56]. Similar to our study, the higher level of RDS in Group H promoted the level of uracil. 5-oxoproline (pyroglutamic acid) could be the intermediate product in the glutathione cycle and its concertation was negatively correlated with the concentration of antioxidant-glutathione [57,58,59]. Based on the correlation analysis, *Lachnospiraceae*_ND3007_group might decrease the concentration of 5-oxoproline and more glutathione was produced in Group H to promote antioxidative capacity. Microbiota degrade dietary protein to tryptophan, which can be later converted into melatonin [60]. Melatonin (N-acetyl-5-methoxytryptamine) as an effective antioxidant [61] could be ultimately oxidized to 5-methoxyindole-3-acetic acid [62]. Hence, the level of 5-methoxyindole-3-acetic acid in Group H with higher RDP density was significantly higher than that in Group C. In this study, catechol as an antioxidant [63,64] was positively related to the relative abundances of *Ruminococcus*_2 and *Prevotella_*1, which might imply that high energy and protein levels in Group H could enhance the catechol concentration by these two genera. Xue et al. [65] reported that the content of spermidine increased in the group of high concentrate diets (rich in starch), which is in line with the results of our study. Furthermore, correlation analysis in this study revealed that spermidine had high positive relationships with *Selenomonas*_1, *Ruminococcaceae*_NK4A214_group, *Lachnospiraceae*_NK3A20_group, *Prevotellaceae_*UCG-004, and *Prevotellaceae_*NK3B31_group. Spermidine is an organic compound widely used as an antioxidant [66]. The upregulation of spermidine observed in Group H of this study might enhance the antioxidative capacity in the rumen of goats by the above genera. These data implied that diets with high energy and protein levels could improve the ruminal antioxidative capacity. However, the potential mechanisms involved in the ruminal antioxidative capacity and the interactions between ruminal bacteria and metabolites are needed to be studied in the future research.

Based on the metabolomic analysis, we found that significantly different metabolites were involved in lipid metabolism and nucleotide metabolism. This result was also identified by PICRUSt2 analysis. Tyrosine metabolism played an important role among those nine main metabolic pathways in this study, which was also detected by Ferguson et al. [67]. Furthermore, the enriched abundances of beta-alanine, arginine, and proline metabolism in this study were related to their functions. Beta-alanine could be metabolized into acetic acid and its concentration is positively associated with the amount of starch and readily available carbohydrate [38]. Arginine and proline involved in RNA synthesis and protein glycosylation are necessary for cellular function [66]. Additionally, we observed that the changes in the concentration of differential metabolites were correlated with pyrimidine metabolism, which was associated with the dietary protein. Dietary nitrogen from protein could be degraded and reused by the microbiota in order to synthesize microbial nucleic acids [38,68]. 

## 5. Conclusions

In this study, 16S rRNA gene sequencing and GC-TOFMS-based metabolomics were used to investigate the changes in rumen bacteria and metabolites in response to the diets with simultaneous changes of dietary energy and protein levels in SWCG. We observed that the bacterial richness was significantly reduced and the rumen bacterial composition was significantly altered with the increasing levels of dietary energy and protein. Metabolomics analysis revealed that the dominant differential metabolites were amino acids, peptides and analogs. Of these, the number of *Prevotella*_1 was significantly increased in the high energy and protein dietary and this genus could promote the catechol synthesis. Moreover, some metabolites could enhance the ruminal antioxidative capacity in Group H, which might modulate the antioxidant activity in the host. In this study, combining with the above results and the previous results of growth performance and carcass characteristics, high energy (ME, 9.24 MJ/kg) and protein (CP, 9.37%) density in diets might be the optimal dietary composition for goats. Moreover, the rumen bacterial community in the solid and epimural fractions of rumen needs to be further studied, which are necessary to gain a more complete understanding of the complexity of the rumen ecosystem [69].

## Figures and Tables

**Table 1:** Composition and nutrient contents of the experimental diets (DM basis).

Items	Treatments	
	Group C	Group H
Ingredients %		
Corn	37.00	34.60
Wheat bran	4.00	5.00
Soybean meal	0.50	0.80
Soybean oil	—	1.50
Corn stalks	55.00	50.00
Alfalfa	—	5.00
Limestone	1.10	0.80
CaHPO_4_·2H_2_O	0.90	0.80
NaCl	0.50	0.50
Premix ^a^	1.00	1.00
Total	100.00	100.00
Nutrient density ^b^		
CP	8.73	9.37
RDP	3.62	4.04
RDS	11.91	12.54
ME (MJ/kg)	8.60	9.24
EE	2.11	3.34
NDF	43.52	42.36
ADF	26.04	25.65

^a^ The premix provides the following per kg of diet: VA 600,000 IU, VD_3_ 200,000 IU, VE 2000 IU, Fe 15 g, Zn 15 g, Cu 4.5 g, I 200 mg and Mn 10 g; ^b^ CP, crude protein; RDP, rumen degradable protein; RDS, rumen degradable starch; ME, metabolic energy; EE, ether extract; NDF, neutral detergent fiber; ADF, acid detergent fiber. The contents of CP, EE, NDF and ADF were measured values, and others were calculated values.

**Table 2:** Analysis of similarities (ANOSIM) for rumen microbial composition at the phylum, genus and OTU level.

Items	*R*			*p* Value		
	Phylum	Genus	OTU	Phylum	Genus	OTU
Groups (H and C)	0.315	0.452	0.426	0.037	0.009	0.014

**Table 3:** Influence of different nutrient density in the diets on the number of bacteria ^a^.

	Groups		SEM	*p* Value ^b^
	**C**	**H**		
Bacteroidetes	6.71	7.60	0.359	0.004
*Prevotella*	6.12	6.88	0.266	0.006

^a^ The number of microbes was shown by log_10_ copies/10 ng DNA. ^b^ All *p-*values were calculated using Student’s *t*-test.

**Table 4:** Significant differential metabolites between Groups C and H (VIP > 1.0; *p* < 0.05).

Metabolite		RT ^a^	Mass	Similarity	VIP	*p* Value	FC ^c^
**Pyridine**							
uracil		11.41	241	889	1.8272	0.026 ^b^	2.044
**Amino acids, peptides, and analogs**							
5-oxoproline		13.80	156	802	1.6496	0.048	0.500
**Fatty acids and conjugates**							
aconitic acid		16.39	229	639	1.9677	0.015	0.563
3,4-dihydroxybenzoic acid		17.17	193	633	1.2278	0.041	0.435
4-hydroxyphenylacetic acid		15.20	179	589	1.2501	0.002	0.332
itaconic acid		11.33	247	478	1.9730	0.041 ^b^	5.630
5-methoxyindole-3-acetic acid		20.62	290	276	1.9622	0.032	88.955
2,4-diaminobutyric acid		15.08	200	261	1.6349	0.021	0.223
2-keto-isovaleric acid		8.22	172	224	1.8180	0.026 ^b^	0.008
**Lipids and lipid-like molecules**							
*O*-phosphoethanolamine		16.75	172	648	1.9346	0.018	0.437
methyl *trans*-cinnamate		12.43	56	274	1.7464	0.017	3.317
**Sugars**							
6-deoxy-D-glucose		16.10	318	458	1.3621	0.015 ^b^	0.199
**Sugar Acids and Derivatives**							
3-phosphoglycerate		16.99	227	546	2.0770	0.026 ^b^	0.009
Amines							
spermidine		20.85	174	577	1.2013	0.038	2.758
**Others**							
	phosphate	10.48	84	758	2.0119	0.002 ^b^	0.068
	pyrophosphate	15.39	451	629	1.9063	0.015 ^b^	0.260
	catechol	11.16	254	472	2.0167	0.002	6.021
	2,8-dihydroxyquinoline	17.46	290	422	1.1676	0.041 ^b^	0.098
	dehydroascorbic acid	17.44	61	307	2.0687	0.026 ^b^	0.001
	tartronic acid	11.876	117	352	1.36	0.041 ^b^	0.213
	isocitric acid	17.139	274	520	1.42	0.009 ^b^	0.010

^a^ Retention time; ^b^ Significant difference between Groups C and H was calculated using Mann-Whitney U. Others were calculated using Student’s *t*-test; ^c^ fold change, FC > 1 means that this metabolite is higher in Group H than that in Group C.

## Supplementary Materials

The following are available online at https://www.mdpi.com/2076-2615/10/7/1193/s1 Table S1. The PCR primers used in this study. Table S2. Number of diversity estimates based on the 16S rRNA gene libraries from the sequencing analysis. Table S3. The phyla of the rumen bacteria in goats. Table S4. Distribution of genera in different groups. Table S5. Predicted functions at level 1 of the rumen bacterial microbiota. Table S6. Predicted functions at level 2 of the rumen bacterial microbiota. Table S7. The mean of relative quantitative values of differential metabolites (VIP > 1.0; *p* < 0.05). Table S8. Sheet 1: The correlation between bacterial genera; sheet 2: The p-value between bacterial genera. Table S9. Sheet 1: The correlation between bacterial genera and differential metabolites; sheet 2: The p-value among bacterial genera and differential metabolites. Figure S1. Metabolic phenotype profile of rumen. (A) GC-TOF/MS total ion current chromatograms of rumen contents from Groups C and H; (B) PCA plot of ruminal metabolites of Groups C and H. Figure S2. Correlation analysis of the top 50 bacterial genera in Group C (A.) and H (B.), respectively. Nodes represent bacterial genera, and edges represent significant interactions among nodes (the absolute Spearman coefficients were above 0.55). The node color corresponds to the phylum taxonomic classification. The edge color represents positive (red) and negative (green) correlations.
