# Supplementary material for: Alterations in Rumen Bacterial Community and Metabolome Characteristics of Cashmere Goats in Response to Dietary Nutrient Density
Source: Animals (Basel). 2020 Jul 14;10(7):1193. doi: 10.3390/ani10071193 (PMC7401628; doi:10.3390/ani10071193)
Supplement: Supplementary file 1 [file animals-10-01193-s001.zip › Supplementary files/Figure S1-S2.docx]

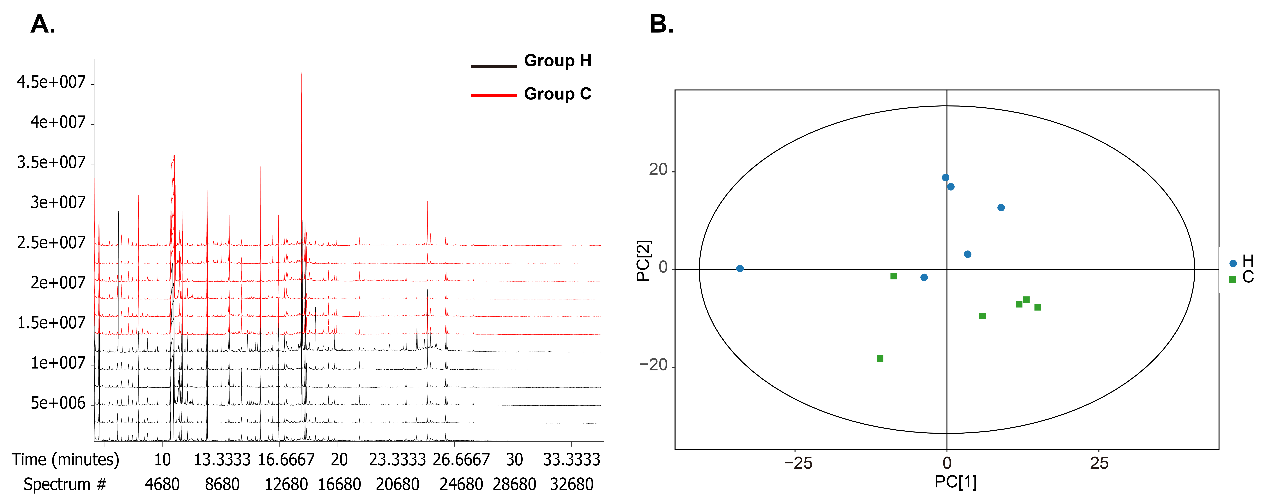


**Figure S1.** Metabolic phenotype profile of rumen. (A) GC-TOF/MS total ion current chromatograms of rumen contents from Groups C and H; (B) PCA plot of ruminal metabolites of Groups C and H.


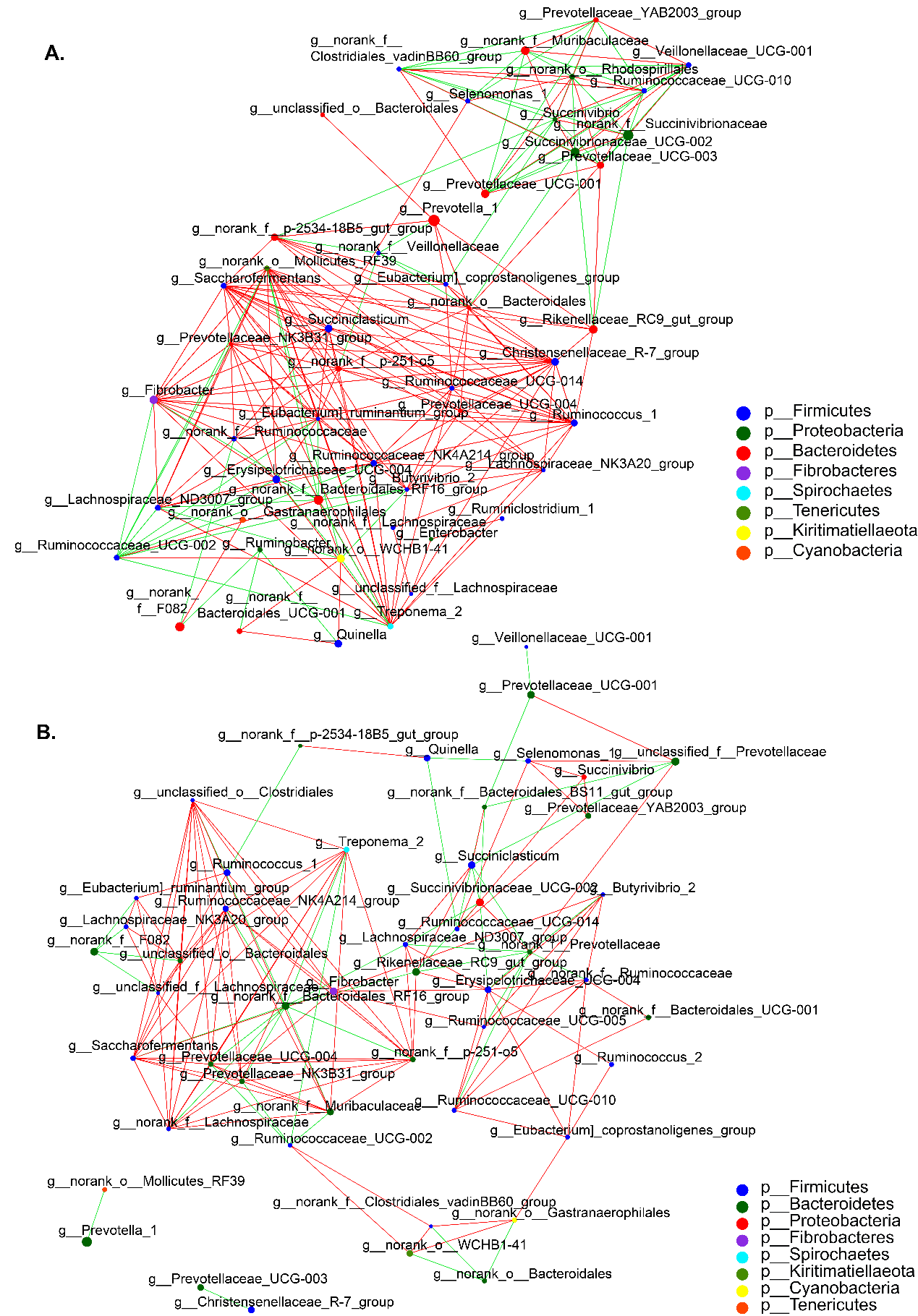


**Figure S2.** Correlation analysis of the top 50 bacterial genera in Group C (A.) or H (B.), respectively. Nodes represent bacterial genera, and edges represent significant interactions among nodes (the absolute Spearman coefficients were above 0.55). The node color corresponds to the phylum taxonomic classification. The edge color represents positive (red) and negative (green) correlations.
