# Supplementary material for: Alterations in Rumen Bacterial Community and Metabolome Characteristics of Cashmere Goats in Response to Dietary Nutrient Density
Source: Animals (Basel). 2020 Jul 14;10(7):1193. doi: 10.3390/ani10071193 (PMC7401628; doi:10.3390/ani10071193)
Supplement: Supplementary file 1 [file animals-10-01193-s001.zip › Supplementary files/Supplementary Tables/Table S1-S7.docx]

**Table S1.** The PCR primers used in this study.

| Target | Primer sequence (5’-3’) *^a^* | Product size (bp) | Annealing temperature (°C) |
| --- | --- | --- | --- |
| *Prevotella* | F: GGTTCTGAGAGGAAGGTCCCC  R: TCCTGCACGCTACTTGGCTG | 121 | 62 [1] |
| Bacteroidetes | F: GGARCATGTGGTTTAATTCGATGAT  R: AGCTGACGACAACCATGCAG | 65 | 60 [2] |

*^a^* F and R indicated forward and reverse primers, respectively.

1. Lettat, A.; Nozière, P.; Silberberg, M.; Morgavi, DP.; Berger, C.; Martin, C. Rumen microbial and fermentation characteristics are affected differently by bacterial probiotic supplementation during induced lactic and subacute acidosis in sheep. *BMC Microbiol.* **2012**, *12*, 142.

2. Garcia-Mazcorro, J.F.; Ivanov, I.; Mills, D.A.; Noratto, G. Influence of whole-wheat consumption on fecal microbial community structure of obese diabetic mice. PEER J. **2016**; *4*, e1702.

**Table S2.** Number of diversity estimates based on the 16S rRNA gene libraries from the sequencing analysis.

| Item | Treatment | | SEM | *p* value |
| --- | --- | --- | --- | --- |
|  | C | H |  |  |
| ACE indices | 1767 | 1564 | 50.908 | 0.038 |
| Chao indices | 1787 | 1575 | 52.664 | 0.036 |
| Shannon indices | 5.174 | 5.285 | 0.136 | 0.703 |
| Simpson indices | 0.032 | 0.020 | 0.006 | 0.896 ^a^ |
| Good’s coverage | 0.995 | 0.996 | 0.000 | 0.031 |

^a^ Significant difference between Groups C and H were calculated using Mann-Whitney U.

Others were calculated using Student’s t-test.

**Table S3.** The phyla in the rumen bacteria of goats.

| Phylum (%) | Treatment | | SEM | *p* value |
| --- | --- | --- | --- | --- |
|  | C | H |  |  |
| Bacteroidetes | 54.46 | 63.76 | 2.155 | 0.022 |
| Firmicutes | 19.04 | 21.20 | 1.651 | 0.873 ^a^ |
| Proteobacteria | 19.13 | 8.40 | 2.718 | 0.041 |
| Fibrobacteres | 2.29 | 2.76 | 0.384 | 0.560 |
| Kiritimatiellaeota | 1.90 | 1.24 | 0.231 | 0.163 |
| Spirochaetae | 0.73 | 0.88 | 0.096 | 0.465 |
| Tenericutes | 0.64 | 0.54 | 0.058 | 0.428 |
| Cyanobacteria | 0.64 | 0.52 | 0.078 | 0.481 |
| Acidobacteria | 0.22 | 0.00 | 0.108 | 0.180 ^a^ |
| Others | 0.96 | 0.68 | 0.153 | 0.392 |

^a^ Significant difference between Groups C and H were calculated using Mann-Whitney U.

Others were calculated using Student’s t-test.

**Table S4.** Distribution of genera in different groups.

| Genus (%) | Treatment | | SEM | *p* value |
| --- | --- | --- | --- | --- |
|  | C | H |  |  |
| *Prevotella*_1 | 25.17 | 35.36 | 2.030 | 0.004 |
| norank_f__*Succinivibrionaceae* | 10.35 | 2.93 | 2.196 | 0.091 |
| unclassified_f__*Prevotellaceae* | 4.85 | 4.53 | 0.411 | 0.720 |
| norank_f__*Bacteroidales*_RF16_group | 5.33 | 3.79 | 0.570 | 0.190 |
| norank_f__F082 | 4.31 | 3.64 | 0.379 | 0.405 |
| *Succinivibrionaceae_*UCG-002 | 3.84 | 3.94 | 1.140 | 0.485 ^a^ |
| *Rikenellaceae_*RC9_gut_group | 2.85 | 3.36 | 0.255 | 0.345 |
| *Prevotellaceae_*UCG-001 | 2.31 | 3.14 | 0.280 | 0.394 ^a^ |
| *Fibrobacter* | 2.28 | 2.76 | 0.384 | 0.555 |
| norank_f__*Muribaculaceae* | 2.51 | 2.03 | 0.307 | 0.458 |
| *Succiniclasticum* | 1.53 | 2.77 | 0.302 | 0.033 |
| *Christensenellaceae_*R-7_group | 1.47 | 1.93 | 0.237 | 0.699 ^a^ |
| *Erysipelotrichaceae_*UCG-004 | 1.72 | 1.57 | 0501 | 0.394 ^a^ |
| norank_o__WCHB1-41 | 1.90 | 1.24 | 0.231 | 0.163 |
| *Quinella* | 1.45 | 1.49 | 0.331 | 0.394 ^a^ |
| *Prevotellaceae*_UCG-003 | 1.11 | 1.43 | 0.081 | 0.044 |
| *Ruminococcus*_1 | 0.92 | 1.57 | 0.184 | 0.180 ^a^ |
| *Ruminococcaceae_*NK4A214_group | 0.67 | 0.95 | 0.092 | 0.139 |
| norank_f__p-2534-18B5_gut_group | 1.14 | 0.29 | 0.245 | 0.818 ^a^ |
| Others | 24.29 | 21.27 | 1.547 | 0.352 |

^a^ Significant difference between Groups C and H were calculated using Mann-Whitney U.

Others were calculated using Student’s t-test.

**Table S5.** Predicted functions at level 1 of the rumen bacterial microbiota.

| Functions (%) | Treatment | | SEM | *p* value |
| --- | --- | --- | --- | --- |
|  | C | H |  |  |
| Metabolism | 50.36 | 50.80 | 0.134 | 0.107 |
| Genetic Information Processing | 20.96 | 21.72 | 0.176 | 0.023 |
| Unclassified | 13.94 | 13.98 | 0.014 | 0.206 |
| Environmental Information Processing | 10.20 | 9.25 | 0.199 | 0.026 ^a^ |
| Cellular Processes | 2.68 | 2.44 | 0.083 | 0.149 |
| Organismal Systems | 0.79 | 0.82 | 0.008 | 0.017 |
| Human Diseases | 0.88 | 0.81 | 0.013 | 0.001 |
| None | 0.18 | 0.19 | 0.001 | 0.475 |

^a^ Significant difference between Groups C and H were calculated using Mann-Whitney U.

Others were calculated using Student’s t-test.

**Table S6.** Predicted functions at level 2 of the rumen bacterial microbiota

| Level 1 | Level 2 | Treatment (%) | | SEM | *p* value ^b^ |
| --- | --- | --- | --- | --- | --- |
|  |  | C | H |  |  |
| Metabolism | Amino acid metabolism | 10.54 | 10.67 | 0.041 | 0.114 |
|  | Carbohydrate metabolism | 9.88 | 9.97 | 0.022 | 0.043 |
|  | Energy metabolism | 6.08 | 6.18 | 0.025 | 0.039 |
|  | Enzyme families | 2.21 | 2.29 | 0.014 | 0.001 |
|  | Glycan biosynthesis and metabolism | 3.20 | 3.30 | 0.056 | 0.390 |
|  | Lipid metabolism | 2.95 | 2.79 | 0.036 | 0.013 |
|  | Metabolism of cofactors and vitamins | 4.84 | 4.95 | 0.039 | 0.178 |
|  | Metabolism of other amino acids | 1.70 | 1.66 | 0.010 | 0.093 |
|  | Metabolism of terpenoids and polyketides | 1.89 | 1.88 | 0.008 | 0.887 |
|  | Nucleotide metabolism | 4.34 | 4.56 | 0.042 | 0.002 |
|  | Xenobiotics biodegradation and metabolism | 1.76 | 1.52 | 0.059 | 0.034 |
|  | Biosynthesis of other secondary metabolites | 1.06 | 1.12 | 0.012 | 0.010 |
| Cellular processes | Cell growth and death | 0.63 | 0.64 | 0.005 | 0.657 |
|  | Cell motility | 1.68 | 1.44 | 0.082 | 0.155 |
| Unclassified | Genetic information processing | 2.61 | 2.63 | 0.012 | 0.257 |
|  | Cellular processes and signaling | 4.01 | 3.98 | 0.020 | 0.536 |
|  | Metabolism | 2.51 | 2.58 | 0.014 | 0.012 |
|  | Poorly characterized | 4.84 | 4.81 | 0.013 | 0.253 |
| Environmental information processing | Membrane transport | 8.62 | 7.85 | 0.166 | 0.012 |
|  | Signal transduction | 1.42 | 1.24 | 0.038 | 0.008 |
| Genetic information processing | Replication and repair | 9.71 | 10.13 | 0.099 | 0.029 |
|  | Transcription | 2.29 | 2.33 | 0.020 | 0.406 |
|  | Translation | 6.23 | 6.51 | 0.063 | 0.017 |
|  | Folding, sorting and degradation | 2.77 | 2.79 | 0.026 | 0.617 |
| Others^a^ | | 2.22 | 2.17 | 0.017 | 0.219 |

^a^ Those with a relative abundance < 0.5 %

^b^ All *p*-value were calculated using Student’s t-test.

**Table S7.** The mean of relative quantitative values of differential metabolites (VIP>1.0; *p*<0.05).

| Metabolite *^a^* | Treatment | | SEM | *p* value |
| --- | --- | --- | --- | --- |
|  | C *^b^* | H *^c^* |  |  |
| uracil | 0.1102 | 0.2252 | 0.02898 | 0.026 ^d^ |
| 5-oxoproline | 2.6100 | 1.3040 | 0.33936 | 0.048 |
| phosphate | 0.1090 | 0.0074 | 0.02219 | 0.002 ^d^ |
| *O*-phosphoethanolamine | 0.0043 | 0.0019 | 0.00055 | 0.018 |
| aconitic acid | 0.0035 | 0.0020 | 0.00034 | 0.015 |
| 3,4-dihydroxybenzoic acid | 0.0251 | 0.0109 | 0.00358 | 0.041 |
| pyrophosphate | 0.0086 | 0.0022 | 0.00132 | 0.015 ^d^ |
| 4-hydroxyphenylacetic acid | 0.0201 | 0.0067 | 0.00255 | 0.002 |
| spermidine | 0.0009 | 0.0025 | 0.00039 | 0.038 |
| 3-phosphoglycerate | 0.0007 | 0.0000 | 0.00015 | 0.026 ^d^ |
| itaconic acid | 0.0001 | 0.0004 | 0.00007 | 0.041 ^d^ |
| catechol | 0.0007 | 0.0041 | 0.00066 | 0.002 |
| 6-deoxy-D-glucose | 0.0034 | 0.0007 | 0.00063 | 0.015 ^d^ |
| 2,8-dihydroxyquinoline | 0.0016 | 0.0002 | 0.00033 | 0.041 ^d^ |
| dehydroascorbic acid | 0.0053 | 0.0000 | 0.00123 | 0.026 ^d^ |
| 5-methoxyindole-3-acetic acid | 0.0000 | 0.0006 | 0.00014 | 0.032 |
| methyl *trans*-cinnamate | 0.0069 | 0.0230 | 0.00360 | 0.017 |
| 2,4-diaminobutyric acid | 0.0334 | 0.0074 | 0.00598 | 0.021 |
| 2-keto-isovaleric acid | 0.0008 | 0.0000 | 0.00018 | 0.026 ^d^ |
| tartronic acid | 0.0043 | 0.0009 | 0.00103 | 0.041 ^d^ |
| isocitric acid | 0.0006 | 0.0000 | 0.00015 | 0.009 ^d^ |

^a^ The name of metabolite;

^b^ The mean of relative quantitative values of metabolites in Group C;

^c^ The mean of relative quantitative values of metabolites in Group H.

^d^ Significant difference between Groups C and H were calculated using Mann-Whitney U.

Others were calculated using Student’s t-test.
